# Diaqua­(nitrato-κ^2^ *O*,*O*′)bis­(l-phenyl­alaninato-κ^2^ *O*,*O*′)lead(II) nitrate

**DOI:** 10.1107/S1600536808006995

**Published:** 2008-03-20

**Authors:** Sylvain Bernès, Laura Gasque

**Affiliations:** a DEP Facultad de Ciencias Químicas, UANL, Guerrero y Progreso S/N, Col. Treviño, 64570 Monterrey, NL, Mexico; b Departamento de Química Inorgánica y Nuclear, Facultad de Química, UNAM, 04510 México, DF, Mexico

## Abstract

In the title complex, [Pb(C_9_H_11_NO_2_)_2_(NO_3_)(H_2_O)_2_]NO_3_, the cation is a monomeric species including zwitterionic amino­acids. In both zwitterions, rotation of the NH_3_^+^ groups about their C—N bonds is blocked by inter­molecular N—H⋯O hydrogen bonds. Assuming a limit for Pb—O bond lengths of 3 Å, the Pb^II^ ion is coordinated by eight O atoms. Each phenyl­alaninate ligand coordinates asymmetrically, with one short and one long Pb—O bond. Coordinated water mol­ecules are also found at significantly different distances, while the bidentate nitrate ion coordinates symmetrically. The resulting [Pb^II^O_8_] core is hemi-directed, with a void placed almost *trans* to a carboxyl­ate group. However, the 6*s* ^2^ lone pair of the metal center can not be considered as stereochemically active, as a non-coordinating O atom of a nitrate belonging to a symmetry-related cation is placed in the empty hemisphere, with a short Pb⋯O separation of 3.035 (10) Å.

## Related literature

A useful classification of Pb complexes into holo- and hemi-directed arrangements of ligands has been proposed by Shimoni-Livny *et al.* (1998 ▶), which allows the prediction of the character of the Pb lone pair. A complex containing zwitterionic phenyl­alaninate has been reported (Apfelbaum-Tibika & Bino, 1984 ▶). Recently, a polymeric Pb^II^ complex of neutral phenyl­alanine has been described (Marandi & Shahbakhsh, 2007 ▶).
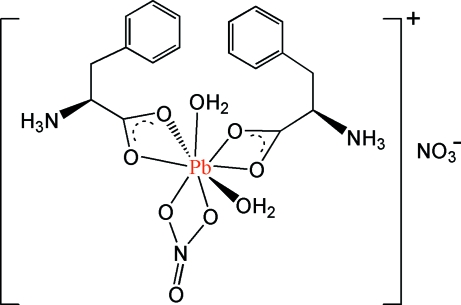


## Experimental

### 

#### Crystal data

[Pb(C_9_H_11_NO_2_)_2_(NO_3_)(H_2_O)_2_]NO_3_
*M* *_r_* = 697.62
Orthorhombic, 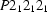

*a* = 5.3851 (9) Å
*b* = 13.5599 (17) Å
*c* = 34.235 (4) Å
*V* = 2499.9 (6) Å^3^
*Z* = 4
Mo *K*α radiation
μ = 6.82 mm^−1^
*T* = 298 (1) K
0.60 × 0.20 × 0.18 mm

#### Data collection

Bruker P4 diffractometer
Absorption correction: ψ scan (*XSCANS*; Siemens, 1996 ▶) *T*_min_ = 0.160, *T*_max_ = 0.294
5243 measured reflections
4948 independent reflections
4026 reflections with *I* > 2σ(*I*)
*R*_int_ = 0.027
3 standard reflections every 97 reflections intensity decay: 1%

#### Refinement

*R*[*F* ^2^ > 2σ(*F* ^2^)] = 0.047
*wR*(*F* ^2^) = 0.110
*S* = 1.06
4948 reflections
319 parameters
18 restraints
H-atom parameters constrained
Δρ_max_ = 1.61 e Å^−3^
Δρ_min_ = −2.35 e Å^−3^
Absolute structure: Flack (1983 ▶), 1153 Friedel pairs
Flack parameter: −0.024 (14)

### 

Data collection: *XSCANS* (Siemens, 1996 ▶); cell refinement: *XSCANS*; data reduction: *XSCANS*; program(s) used to solve structure: *SHELXTL-Plus* (Sheldrick, 2008 ▶); program(s) used to refine structure: *SHELXTL-Plus*; molecular graphics: *SHELXTL-Plus* and *Mercury* (Macrae *et al.*, 2006 ▶); software used to prepare material for publication: *SHELXTL-Plus*.

## Supplementary Material

Crystal structure: contains datablocks I, global. DOI: 10.1107/S1600536808006995/ez2121sup1.cif

Structure factors: contains datablocks I. DOI: 10.1107/S1600536808006995/ez2121Isup2.hkl

Additional supplementary materials:  crystallographic information; 3D view; checkCIF report

## Figures and Tables

**Table 1:** Selected bond lengths (Å)

Pb1—O1	2.354 (7)
Pb1—O2	2.979 (6)
Pb1—O11	2.453 (7)
Pb1—O12	2.791 (7)
Pb1—O3	2.628 (8)
Pb1—O4	2.886 (6)
Pb1—O21	2.927 (12)
Pb1—O22	2.994 (11)

**Table 2:** Hydrogen-bond geometry (Å, °)

*D*—H⋯*A*	*D*—H	H⋯*A*	*D*⋯*A*	*D*—H⋯*A*
N1—H1*A*⋯O4^i^	0.92	2.25	2.956 (11)	134
N1—H1*B*⋯O33	0.92	2.07	2.932 (11)	155
N1—H1*C*⋯O32^ii^	0.92	1.87	2.786 (11)	173
N11—H11*A*⋯O22^iii^	0.92	1.95	2.800 (12)	152
N11—H11*B*⋯O33^iv^	0.92	1.95	2.749 (9)	145
N11—H11*C*⋯O12^v^	0.92	1.96	2.833 (11)	158

Symmetry codes: (i) 
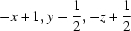
; (ii) 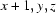; (iii) 
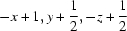
; (iv) 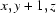; (v) 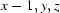.

**Fig. 1.**
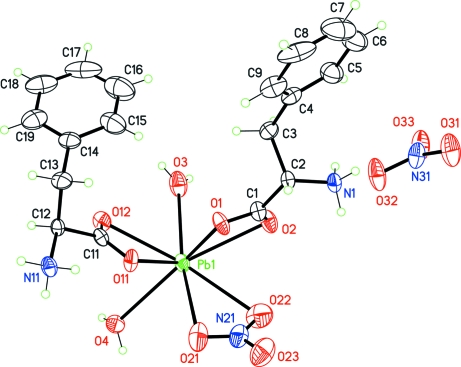
The asymmetric unit of (I) showing the atom numbering scheme, with displacement ellipsoids at the 30% probability level.

**Fig. 2.**
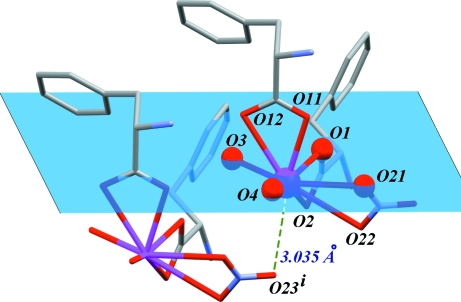
Two symmetry-related molecules in the crystal structure, omitting H atoms and non-coordinated nitrate ions. The blue plane is calculated using atoms Pb1/O1/O3/O4/O21 (spheres of arbitrary radii) and corresponds to the equatorial plane defining the hemidirected ligand arrangement. The short inter-cation contact is shown as a dashed line. Symmetry code: (i) 1 + x, y, z.

## supplementary crystallographic information

### Comment

A particular feature characterizing Pb^II^ is the role of the 6*s*^2^ lone pair, which determines the arrangement of ligands coordinating to this metal ion. A stereochemically active lone pair is reflected in a hemidirected disposition of ligands around Pb^II^, *i.e.* with all coordination bonds directed throughout only one hemisphere of an encompassing globe. In such a case, the lone pair is assumed to be localized in the empty hemisphere. The opposite situation is found in holodirected Pb^II^ complexes, in which the bonds to ligand atoms are distributed isotropically around the metal center (Shimoni-Livny *et al.*, 1998). For coordination number 8, examples of either type of stereochemistry have been found. The complex reported here, (I), may be considered as a borderline case: although the coordination geometry seems to be hemidirected, the lone pair is probably not stereochemically active.

Complex (I) was prepared and crystallized from water (see *Experimental*) and is a part of our general research dealing with interactions between aminoacids and Pb^II^. The naturally occurring aminoacid *L*-phenlyalanine is found in its zwitterionic form, and coordinates through O atoms of the carboxylate group. The coordination is asymmetric, however, all carboxylic O atoms may be considered as bonded to the metal ion, assuming a limit for Pb—O bond lengths of 3 Å. To date, only one complex that includes zwitterionic phenylalaninate has been characterized by X-ray diffraction (Apfelbaum-Tibika & Bino, 1984). This aminoacid, in its neutral form, was recently coordinated to Pb^II^, forming a polymeric structure (Marandi & Shahbakhsh, 2007). In (I), rotation of NH_3_^+^ groups about their C—N bonds is blocked by intermolecular N—H···O hydrogen bonds, involving nitrate and water O atoms. The cation is completed by two water molecules, found at significantly different distances, and a bidentate nitrate ion, which coordinates in a symmetric manner. The asymmetric unit contains one cation and one nitrate counter-ion (Fig. 1).

The [Pb^II^O_8_] core structure in the cation is best described as hemidirected. The equatorial plane defining the encompassing globe contains atoms Pb1/O1/O3/O4/O21 (Fig. 2, top molecule). Although atoms O2 and O22 are placed in the *anti*-hemisphere, they are close to the equatorial plane, since they belong to carboxylate groups forming bite angles [O1—Pb1—O2: 47.3 (2)°; O21—Pb1—O22: 42.6 (2)]. Deviations of these atoms from the mean plane Pb1/O1/O3/O4/O21 are 1.81 (O2) and 1.84 Å (O22). This arrangement for O atoms around Pb^II^ is thus consistent with the presence of a stereochemically active lone pair 6*s*^2^, placed roughly *trans* to the carboxylate group O11/O12. However, a careful examination of the packing structure reveals a short Pb···O intermolecular contact, involving a nitrate group of a neighboring cation (Fig. 2, bottom molecule). The contact distance is sufficiently short, 3.035 (10) Å, to impede activity for the lone pair, which is thus expected to have little *p* character.

### Experimental

An amount of Pb(NO_3_)_2_ (1 mmol, 0.331 g) was dissolved in 80 ml of previously degassed and distilled water. Solid *L*-phenylalanine (2 mmol, 0.330 g, Sigma) was added in small fractions with magnetic stirring, dissolving upon coordination to the metal ion. After the complete addition with continuous stirring, pH was adjusted to 5.3 by dropwise addition of 0.01 *M* NaOH. The solution was left to rest, and colourless needles were collected after one week.

### Refinement

In order to avoid too large differences between displacement parameters in the coordinated nitrate anion, atoms N21, O21, O22 and O23 were restrained, with a standard deviation of 0.01 Å^2^, to have the same *U*_ij_ components. Almost all H atoms were detected in a difference map. They were however placed in idealized positions, and refined as riding to their parent atoms. Bond lengths were fixed to 0.85 (OH), 0.92 (NH), 0.93 (aromatic CH), 0.97 (methylene CH_2_) and 0.98 Å (methine CH). Isotropic displacement parameters were calculated as *U*_iso_(H) = 1.2 *U*_eq_(carrier C) for C-bonded H atoms, and *U*_iso_(H) = 1.5 *U*_eq_(carrier atom) otherwise. Rigid NH_3_ groups were allowed to rotate about their C—N bonds.

### Crystal data

**Table:**

[Pb(C_9_H_11_NO_2_)_2_(NO_3_)(H_2_O)_2_]NO_3_	*D*_x_ = 1.854 Mg m^−^^3^
*M**_r_* = 697.62	Mo *K*α radiation λ = 0.71073 Å
Orthorhombic, *P*2_1_2_1_2_1_	Cell parameters from 83 reflections
Hall symbol: P 2ac 2ab	θ = 4.7–14.1º
*a* = 5.3851 (9) Å	µ = 6.82 mm^−^^1^
*b* = 13.5599 (17) Å	*T* = 298 (1) K
*c* = 34.235 (4) Å	Cell measurement pressure: 101(1) kPa
*V* = 2499.9 (6) Å^3^	Needle, colourless
*Z* = 4	0.60 × 0.20 × 0.18 mm
*F*_000_ = 1360	

### Data collection

**Table:**

Bruker P4 diffractometer	4026 reflections with *I* > 2σ(*I*)
Radiation source: fine-focus sealed tube	*R*_int_ = 0.027
Monochromator: graphite	θ_max_ = 29.0º
*T* = 298(1) K	θ_min_ = 1.6º
*P* = 101(1) kPa	*h* = −7→2
ω scans	*k* = −1→18
Absorption correction: ψ scan(XSCANS; Siemens, 1996)	*l* = −1→46
*T*_min_ = 0.160, *T*_max_ = 0.294	3 standard reflections
5243 measured reflections	every 97 reflections
4948 independent reflections	intensity decay: 1%

### Refinement

**Table:**

Refinement on *F*^2^	Hydrogen site location: inferred from neighbouring sites
Least-squares matrix: full	H-atom parameters constrained
*R*[*F*^2^ > 2σ(*F*^2^)] = 0.047	*w* = 1/[σ^2^(*F*_o_^2^) + (0.044*P*)^2^ + 6.7647*P*] where *P* = (*F*_o_^2^ + 2*F*_c_^2^)/3
*wR*(*F*^2^) = 0.110	(Δ/σ)_max_ = 0.001
*S* = 1.06	Δρ_max_ = 1.61 e Å^−^^3^
4948 reflections	Δρ_min_ = −2.35 e Å^−^^3^
319 parameters	Extinction correction: SHELXTL-Plus (Sheldrick, 2008), Fc^*^=kFc[1+0.001xFc^2^λ^3^/sin(2θ)]^-1/4^
18 restraints	Extinction coefficient: 0.0034 (3)
Primary atom site location: structure-invariant direct methods	Absolute structure: Flack (1983), 1153 Friedel pairs
Secondary atom site location: difference Fourier map	Flack parameter: −0.024 (14)

### Fractional atomic coordinates and isotropic or equivalent isotropic displacement parameters (Å^2^)

**Table:**

	*x*	*y*	*z*	*U*_iso_*/*U*_eq_	
Pb1	0.77229 (7)	0.50814 (2)	0.280652 (9)	0.04579 (14)	
C1	0.586 (2)	0.3234 (7)	0.3200 (3)	0.044 (2)	
N1	0.4874 (15)	0.1511 (5)	0.3341 (2)	0.0444 (18)	
H1A	0.4767	0.1481	0.3074	0.067*	
H1B	0.3683	0.1109	0.3450	0.067*	
H1C	0.6423	0.1303	0.3420	0.067*	
O1	0.5070 (14)	0.4129 (4)	0.3202 (2)	0.0543 (19)	
C2	0.4473 (19)	0.2538 (6)	0.3472 (3)	0.040 (2)	
H2A	0.2695	0.2689	0.3460	0.047*	
O2	0.7618 (16)	0.2944 (4)	0.3007 (2)	0.063 (2)	
C3	0.542 (2)	0.2689 (7)	0.3900 (3)	0.049 (2)	
H3A	0.7103	0.2441	0.3920	0.059*	
H3B	0.5457	0.3389	0.3957	0.059*	
C4	0.383 (2)	0.2178 (8)	0.4199 (3)	0.052 (3)	
C5	0.424 (3)	0.1204 (9)	0.4308 (3)	0.072 (3)	
H5A	0.5568	0.0861	0.4199	0.086*	
C6	0.274 (3)	0.0743 (11)	0.4570 (4)	0.092 (4)	
H6A	0.3058	0.0091	0.4638	0.110*	
C7	0.081 (4)	0.1218 (18)	0.4732 (4)	0.113 (8)	
H7A	−0.0191	0.0892	0.4912	0.135*	
C8	0.030 (3)	0.2169 (18)	0.4634 (4)	0.105 (7)	
H8A	−0.1051	0.2494	0.4746	0.127*	
C9	0.182 (2)	0.2651 (10)	0.4365 (3)	0.067 (3)	
H9A	0.1476	0.3300	0.4296	0.080*	
C11	0.5983 (19)	0.6746 (6)	0.3283 (3)	0.037 (2)	
N11	0.2477 (15)	0.7919 (4)	0.3286 (2)	0.0433 (16)	
H11A	0.2942	0.8134	0.3042	0.065*	
H11B	0.1714	0.8425	0.3419	0.065*	
H11C	0.1392	0.7399	0.3262	0.065*	
O11	0.4650 (14)	0.6227 (4)	0.3079 (2)	0.0532 (18)	
C12	0.4715 (18)	0.7594 (6)	0.3506 (2)	0.035 (2)	
H12A	0.5875	0.8148	0.3527	0.042*	
O12	0.8305 (13)	0.6650 (5)	0.3327 (2)	0.0514 (18)	
C13	0.390 (2)	0.7277 (8)	0.3918 (2)	0.049 (2)	
H13A	0.3070	0.7829	0.4043	0.059*	
H13B	0.2697	0.6747	0.3895	0.059*	
C14	0.597 (2)	0.6940 (8)	0.4175 (3)	0.055 (3)	
C15	0.644 (3)	0.5949 (10)	0.4225 (4)	0.085 (4)	
H15A	0.5448	0.5489	0.4099	0.102*	
C16	0.837 (4)	0.5621 (15)	0.4461 (5)	0.119 (6)	
H16A	0.8706	0.4951	0.4484	0.143*	
C17	0.979 (4)	0.631 (2)	0.4661 (4)	0.117 (8)	
H17A	1.1048	0.6092	0.4826	0.140*	
C18	0.936 (3)	0.7289 (17)	0.4620 (4)	0.096 (6)	
H18A	1.0341	0.7749	0.4750	0.115*	
C19	0.742 (3)	0.7592 (10)	0.4377 (3)	0.075 (3)	
H19A	0.7103	0.8264	0.4352	0.090*	
O3	1.0478 (14)	0.4723 (5)	0.3424 (3)	0.076 (2)	
H31	1.0178	0.5014	0.3639	0.114*	
H32	1.1827	0.4402	0.3402	0.114*	
O4	0.8220 (16)	0.6871 (5)	0.23573 (17)	0.0556 (18)	
H41	0.7685	0.6839	0.2124	0.083*	
H42	0.9634	0.7148	0.2371	0.083*	
N21	0.290 (2)	0.4320 (7)	0.2286 (3)	0.069 (2)	
O21	0.314 (2)	0.5177 (6)	0.2350 (3)	0.095 (2)	
O22	0.465 (2)	0.3712 (8)	0.2319 (3)	0.097 (3)	
O23	0.0891 (19)	0.3971 (8)	0.2220 (3)	0.094 (3)	
N31	−0.0031 (15)	0.0127 (6)	0.3617 (3)	0.057 (2)	
O31	−0.1845 (18)	−0.0373 (6)	0.3673 (3)	0.089 (3)	
O32	−0.0270 (14)	0.1025 (5)	0.3546 (3)	0.075 (3)	
O33	0.2098 (14)	−0.0227 (5)	0.3605 (3)	0.074 (2)	

### Atomic displacement parameters (Å^2^)

**Table:**

	*U*^11^	*U*^22^	*U*^33^	*U*^12^	*U*^13^	*U*^23^
Pb1	0.0489 (2)	0.03533 (16)	0.05312 (19)	0.00897 (17)	0.01081 (17)	−0.00091 (13)
C1	0.035 (6)	0.038 (4)	0.057 (6)	0.001 (4)	0.009 (5)	0.000 (4)
N1	0.044 (5)	0.030 (3)	0.059 (5)	0.000 (3)	−0.003 (4)	−0.001 (3)
O1	0.047 (4)	0.033 (3)	0.082 (5)	0.011 (3)	0.017 (4)	0.012 (3)
C2	0.038 (6)	0.030 (4)	0.051 (5)	−0.004 (4)	0.004 (5)	0.000 (4)
O2	0.065 (5)	0.043 (3)	0.080 (4)	0.001 (4)	0.029 (5)	0.006 (3)
C3	0.043 (6)	0.055 (5)	0.047 (5)	0.001 (5)	−0.004 (5)	−0.006 (4)
C4	0.049 (6)	0.066 (6)	0.041 (5)	−0.007 (5)	−0.005 (5)	−0.009 (4)
C5	0.080 (9)	0.072 (7)	0.064 (7)	0.004 (7)	0.009 (7)	0.006 (6)
C6	0.107 (13)	0.100 (9)	0.068 (7)	−0.026 (12)	0.000 (9)	0.022 (7)
C7	0.089 (14)	0.18 (2)	0.073 (10)	−0.043 (16)	0.007 (9)	0.033 (12)
C8	0.069 (11)	0.21 (2)	0.042 (7)	0.006 (14)	0.005 (7)	−0.022 (10)
C9	0.048 (7)	0.096 (8)	0.057 (6)	0.019 (7)	0.002 (6)	−0.013 (5)
C11	0.028 (5)	0.031 (4)	0.051 (5)	0.001 (4)	0.006 (4)	0.008 (4)
N11	0.036 (4)	0.034 (3)	0.060 (4)	0.007 (4)	0.001 (4)	0.001 (3)
O11	0.049 (5)	0.036 (3)	0.075 (5)	0.001 (3)	−0.002 (4)	−0.016 (3)
C12	0.035 (5)	0.028 (3)	0.043 (5)	0.005 (4)	0.001 (4)	−0.003 (3)
O12	0.041 (4)	0.051 (4)	0.062 (4)	0.020 (3)	0.009 (3)	−0.001 (3)
C13	0.049 (6)	0.061 (6)	0.037 (4)	0.006 (5)	0.013 (5)	0.002 (4)
C14	0.054 (7)	0.071 (7)	0.039 (5)	0.005 (6)	0.011 (5)	0.007 (5)
C15	0.089 (11)	0.088 (9)	0.078 (8)	0.022 (9)	0.005 (8)	0.021 (7)
C16	0.125 (16)	0.128 (14)	0.103 (12)	0.057 (14)	0.013 (12)	0.032 (11)
C17	0.088 (13)	0.21 (3)	0.049 (8)	0.052 (16)	0.009 (7)	0.029 (11)
C18	0.068 (11)	0.167 (18)	0.052 (8)	−0.016 (12)	0.004 (7)	0.009 (9)
C19	0.069 (9)	0.104 (8)	0.052 (6)	−0.019 (9)	0.014 (7)	0.000 (5)
O3	0.053 (4)	0.051 (4)	0.124 (7)	0.006 (4)	−0.001 (5)	−0.004 (5)
O4	0.064 (5)	0.057 (4)	0.046 (3)	−0.009 (4)	0.014 (4)	0.007 (3)
N21	0.077 (5)	0.058 (4)	0.071 (4)	−0.001 (4)	0.004 (5)	−0.015 (3)
O21	0.109 (6)	0.062 (4)	0.113 (5)	−0.016 (5)	0.013 (5)	−0.018 (4)
O22	0.097 (7)	0.108 (6)	0.086 (5)	0.035 (6)	0.003 (5)	−0.019 (5)
O23	0.076 (5)	0.108 (6)	0.099 (6)	−0.032 (5)	0.009 (5)	−0.021 (5)
N31	0.046 (5)	0.042 (4)	0.083 (6)	−0.002 (4)	0.004 (4)	−0.017 (4)
O31	0.069 (5)	0.059 (4)	0.137 (7)	−0.024 (5)	0.011 (6)	0.003 (5)
O32	0.041 (4)	0.033 (3)	0.151 (8)	0.007 (3)	−0.002 (5)	0.002 (4)
O33	0.042 (4)	0.041 (3)	0.138 (7)	0.011 (4)	−0.006 (4)	−0.015 (4)

### Geometric parameters (Å, °)

**Table:**

Pb1—O1	2.354 (7)	C11—O12	1.266 (12)
Pb1—O2	2.979 (6)	C11—C12	1.540 (12)
Pb1—O11	2.453 (7)	N11—C12	1.488 (11)
Pb1—O12	2.791 (7)	N11—H11A	0.9200
Pb1—O3	2.628 (8)	N11—H11B	0.9200
Pb1—O4	2.886 (6)	N11—H11C	0.9200
Pb1—O21	2.927 (12)	C12—C13	1.541 (12)
Pb1—O22	2.994 (11)	C12—H12A	0.9800
C1—O2	1.217 (12)	C13—C14	1.490 (15)
C1—O1	1.287 (11)	C13—H13A	0.9700
C1—C2	1.522 (13)	C13—H13B	0.9700
N1—C2	1.479 (10)	C14—C19	1.370 (16)
N1—H1A	0.9200	C14—C15	1.380 (16)
N1—H1B	0.9200	C15—C16	1.39 (2)
N1—H1C	0.9200	C15—H15A	0.9300
C2—C3	1.566 (13)	C16—C17	1.38 (3)
C2—H2A	0.9800	C16—H16A	0.9300
C3—C4	1.503 (15)	C17—C18	1.36 (3)
C3—H3A	0.9700	C17—H17A	0.9300
C3—H3B	0.9700	C18—C19	1.40 (2)
C4—C9	1.379 (15)	C18—H18A	0.9300
C4—C5	1.390 (16)	C19—H19A	0.9300
C5—C6	1.359 (18)	O3—H31	0.8500
C5—H5A	0.9300	O3—H32	0.8500
C6—C7	1.34 (3)	O4—H41	0.8501
C6—H6A	0.9300	O4—H42	0.8500
C7—C8	1.36 (3)	N21—O21	1.189 (11)
C7—H7A	0.9300	N21—O23	1.203 (14)
C8—C9	1.40 (2)	N21—O22	1.257 (13)
C8—H8A	0.9300	N31—O31	1.204 (11)
C9—H9A	0.9300	N31—O33	1.243 (11)
C11—O11	1.225 (12)	N31—O32	1.249 (11)
			
O1—Pb1—O11	73.7 (2)	C8—C7—H7A	119.7
O1—Pb1—O3	77.2 (3)	C7—C8—C9	119.1 (18)
O11—Pb1—O3	101.1 (2)	C7—C8—H8A	120.5
O1—Pb1—O12	96.8 (2)	C9—C8—H8A	120.5
O11—Pb1—O12	49.5 (2)	C4—C9—C8	121.0 (14)
O3—Pb1—O12	64.2 (2)	C4—C9—H9A	119.5
O1—Pb1—O4	145.5 (2)	C8—C9—H9A	119.5
O11—Pb1—O4	74.5 (2)	O11—C11—O12	126.0 (10)
O3—Pb1—O4	122.1 (2)	O11—C11—C12	116.9 (9)
O12—Pb1—O4	71.87 (19)	O12—C11—C12	117.2 (10)
O1—Pb1—O21	79.6 (3)	C12—N11—H11A	109.5
O11—Pb1—O21	66.8 (2)	C12—N11—H11B	109.5
O3—Pb1—O21	156.2 (3)	H11A—N11—H11B	109.5
O12—Pb1—O21	113.7 (2)	C12—N11—H11C	109.5
O4—Pb1—O21	75.9 (2)	H11A—N11—H11C	109.5
O1—Pb1—O2	47.3 (2)	H11B—N11—H11C	109.5
O11—Pb1—O2	121.0 (2)	C11—O11—Pb1	100.7 (6)
O3—Pb1—O2	69.2 (2)	N11—C12—C13	108.4 (8)
O12—Pb1—O2	126.6 (2)	N11—C12—C11	109.3 (7)
O4—Pb1—O2	160.55 (19)	C13—C12—C11	111.9 (7)
O21—Pb1—O2	98.6 (2)	N11—C12—H12A	109.1
O1—Pb1—O22	69.2 (3)	C13—C12—H12A	109.1
O11—Pb1—O22	103.4 (3)	C11—C12—H12A	109.1
O3—Pb1—O22	130.2 (3)	C11—O12—Pb1	83.8 (7)
O12—Pb1—O22	152.9 (2)	C14—C13—C12	114.3 (9)
O4—Pb1—O22	106.0 (3)	C14—C13—H13A	108.7
O21—Pb1—O22	42.6 (2)	C12—C13—H13A	108.7
O2—Pb1—O22	61.0 (3)	C14—C13—H13B	108.7
O2—C1—O1	124.4 (9)	C12—C13—H13B	108.7
O2—C1—C2	120.9 (8)	H13A—C13—H13B	107.6
O1—C1—C2	114.7 (9)	C19—C14—C15	117.3 (12)
C2—N1—H1A	109.5	C19—C14—C13	121.9 (11)
C2—N1—H1B	109.5	C15—C14—C13	120.8 (12)
H1A—N1—H1B	109.5	C14—C15—C16	121.6 (17)
C2—N1—H1C	109.5	C14—C15—H15A	119.2
H1A—N1—H1C	109.5	C16—C15—H15A	119.2
H1B—N1—H1C	109.5	C17—C16—C15	119.1 (18)
C1—O1—Pb1	108.2 (6)	C17—C16—H16A	120.5
N1—C2—C1	109.1 (7)	C15—C16—H16A	120.5
N1—C2—C3	111.0 (8)	C18—C17—C16	120.9 (19)
C1—C2—C3	109.3 (8)	C18—C17—H17A	119.6
N1—C2—H2A	109.1	C16—C17—H17A	119.6
C1—C2—H2A	109.1	C17—C18—C19	118.5 (18)
C3—C2—H2A	109.1	C17—C18—H18A	120.8
C1—O2—Pb1	80.0 (5)	C19—C18—H18A	120.8
C4—C3—C2	113.2 (9)	C14—C19—C18	122.6 (14)
C4—C3—H3A	108.9	C14—C19—H19A	118.7
C2—C3—H3A	108.9	C18—C19—H19A	118.7
C4—C3—H3B	108.9	Pb1—O3—H31	120.1
C2—C3—H3B	108.9	Pb1—O3—H32	120.5
H3A—C3—H3B	107.8	H31—O3—H32	118.4
C9—C4—C5	117.2 (12)	Pb1—O4—H41	115.2
C9—C4—C3	120.8 (11)	Pb1—O4—H42	115.0
C5—C4—C3	121.9 (11)	H41—O4—H42	112.3
C6—C5—C4	121.3 (13)	O21—N21—O23	120.9 (13)
C6—C5—H5A	119.4	O21—N21—O22	123.0 (14)
C4—C5—H5A	119.4	O23—N21—O22	115.8 (10)
C7—C6—C5	120.8 (16)	N21—O21—Pb1	98.3 (9)
C7—C6—H6A	119.6	N21—O22—Pb1	93.3 (7)
C5—C6—H6A	119.6	O31—N31—O33	122.4 (9)
C6—C7—C8	120.7 (17)	O31—N31—O32	119.8 (9)
C6—C7—H7A	119.7	O33—N31—O32	117.7 (9)
			
O2—C1—O1—Pb1	−3.4 (14)	O11—C11—C12—N11	−28.1 (11)
C2—C1—O1—Pb1	174.9 (7)	O12—C11—C12—N11	151.7 (9)
O11—Pb1—O1—C1	−177.6 (8)	O11—C11—C12—C13	91.9 (11)
O3—Pb1—O1—C1	−71.9 (7)	O12—C11—C12—C13	−88.3 (11)
O12—Pb1—O1—C1	−133.3 (7)	O11—C11—O12—Pb1	0.7 (11)
O4—Pb1—O1—C1	158.9 (6)	C12—C11—O12—Pb1	−179.1 (7)
O21—Pb1—O1—C1	113.7 (7)	O1—Pb1—O12—C11	−62.1 (6)
O2—Pb1—O1—C1	1.6 (6)	O11—Pb1—O12—C11	−0.4 (6)
O22—Pb1—O1—C1	70.7 (7)	O3—Pb1—O12—C11	−134.3 (7)
O2—C1—C2—N1	−22.5 (14)	O4—Pb1—O12—C11	84.4 (6)
O1—C1—C2—N1	159.1 (9)	O21—Pb1—O12—C11	19.5 (7)
O2—C1—C2—C3	99.1 (11)	O2—Pb1—O12—C11	−102.5 (6)
O1—C1—C2—C3	−79.4 (12)	O22—Pb1—O12—C11	−5.4 (9)
O1—C1—O2—Pb1	2.6 (11)	N11—C12—C13—C14	−179.4 (8)
C2—C1—O2—Pb1	−175.7 (10)	C11—C12—C13—C14	60.0 (11)
O1—Pb1—O2—C1	−1.6 (7)	C12—C13—C14—C19	82.9 (12)
O11—Pb1—O2—C1	−0.7 (7)	C12—C13—C14—C15	−99.1 (12)
O3—Pb1—O2—C1	90.0 (7)	C19—C14—C15—C16	−2.2 (19)
O12—Pb1—O2—C1	59.6 (7)	C13—C14—C15—C16	179.6 (12)
O4—Pb1—O2—C1	−140.7 (8)	C14—C15—C16—C17	2(2)
O21—Pb1—O2—C1	−68.7 (7)	C15—C16—C17—C18	−2(3)
O22—Pb1—O2—C1	−89.3 (7)	C16—C17—C18—C19	1(3)
N1—C2—C3—C4	−70.6 (11)	C15—C14—C19—C18	1.6 (18)
C1—C2—C3—C4	169.0 (9)	C13—C14—C19—C18	179.8 (11)
C2—C3—C4—C9	−89.2 (11)	C17—C18—C19—C14	−1(2)
C2—C3—C4—C5	87.8 (13)	O23—N21—O21—Pb1	155.0 (10)
C9—C4—C5—C6	−0.8 (18)	O22—N21—O21—Pb1	−18.1 (12)
C3—C4—C5—C6	−177.9 (11)	O1—Pb1—O21—N21	−61.2 (8)
C4—C5—C6—C7	0(2)	O11—Pb1—O21—N21	−137.8 (8)
C5—C6—C7—C8	1(2)	O3—Pb1—O21—N21	−74.7 (9)
C6—C7—C8—C9	0(2)	O12—Pb1—O21—N21	−154.2 (7)
C5—C4—C9—C8	0.9 (17)	O4—Pb1—O21—N21	143.3 (8)
C3—C4—C9—C8	178.1 (11)	O2—Pb1—O21—N21	−17.7 (8)
C7—C8—C9—C4	0(2)	O22—Pb1—O21—N21	9.3 (7)
O12—C11—O11—Pb1	−0.8 (12)	O21—N21—O22—Pb1	17.6 (12)
C12—C11—O11—Pb1	179.0 (6)	O23—N21—O22—Pb1	−155.9 (9)
O1—Pb1—O11—C11	114.7 (6)	O1—Pb1—O22—N21	88.6 (7)
O3—Pb1—O11—C11	41.7 (6)	O11—Pb1—O22—N21	22.1 (7)
O12—Pb1—O11—C11	0.4 (6)	O3—Pb1—O22—N21	139.5 (7)
O4—Pb1—O11—C11	−78.8 (6)	O12—Pb1—O22—N21	26.0 (11)
O21—Pb1—O11—C11	−159.8 (7)	O4—Pb1—O22—N21	−55.3 (7)
O2—Pb1—O11—C11	114.0 (6)	O21—Pb1—O22—N21	−8.7 (6)
O22—Pb1—O11—C11	178.0 (6)	O2—Pb1—O22—N21	140.4 (8)

### Hydrogen-bond geometry (Å, °)

**Table:**

*D*—H···*A*	*D*—H	H···*A*	*D*···*A*	*D*—H···*A*
N1—H1A···O4^i^	0.92	2.25	2.956 (11)	134
N1—H1B···O33	0.92	2.07	2.932 (11)	155
N1—H1C···O32^ii^	0.92	1.87	2.786 (11)	173
N11—H11A···O22^iii^	0.92	1.95	2.800 (12)	152
N11—H11B···O33^iv^	0.92	1.95	2.749 (9)	145
N11—H11C···O12^v^	0.92	1.96	2.833 (11)	158
O3—H32···O1^ii^	0.85	1.91	2.710 (11)	156
O4—H42···O2^vi^	0.85	2.24	2.949 (10)	140

Symmetry codes: (i) −*x*+1, *y*−1/2, −*z*+1/2; (ii) *x*+1, *y*, *z*; (iii) −*x*+1, *y*+1/2, −*z*+1/2; (iv) *x*, *y*+1, *z*; (v) *x*−1, *y*, *z*; (vi) −*x*+2, *y*+1/2, −*z*+1/2.
